# Costs and economies of scale in repeated home-based HIV counselling and testing: Evidence from the ANRS 12249 Treatment as Prevention trial in South Africa

**DOI:** 10.1016/j.socscimed.2022.115068

**Published:** 2022-07

**Authors:** Marwân-al-Qays Bousmah, Collins Iwuji, Nonhlanhla Okesola, Joanna Orne-Gliemann, Deenan Pillay, François Dabis, Joseph Larmarange, Sylvie Boyer, for the ANRS 12249 TasP Study Group

**Affiliations:** a Aix Marseille Univ, Inserm, IRD, SESSTIM, Sciences Economiques & Sociales de la Santé & Traitement de l'Information Médicale, ISSPAM, Marseille, France; b Université Paris Cité, IRD, Inserm, Ceped, F-75006, Paris, France; c Africa Health Research Institute, Durban, KwaZulu-Natal, South Africa; d Department of Global Health and Infection, Brighton and Sussex Medical School, University of Sussex, Brighton, UK; e University of Bordeaux, National Institute for Health and Medical Research (INSERM) UMR 1219, Research Institute for Sustainable Development (IRD) EMR 271, Bordeaux Population Health Centre, Bordeaux, France; f Research Department of Infection and Population Health, University College London, London, UK; g Division of Infection and Immunity, University College London, London, UK

**Keywords:** AIDS/HIV, Prevention, Cost of care, Economies of scale, Interventions, Clinical trials, South Africa

## Abstract

Universal HIV testing is now recommended in generalised HIV epidemic settings. Although home-based HIV counselling and testing (HB-HCT) has been shown to be effective in achieving high levels of HIV status awareness, little is still known about the cost implications of universal and repeated HB-HCT. We estimated the costs of repeated HB-HCT and the scale economies that can be obtained when increasing the population coverage of the intervention. We used primary data from the ANRS 12249 Treatment as Prevention (TasP) trial in rural South Africa (2012–2016), whose testing component included six-monthly repeated HB-HCT. We relied on the dynamic system generalised method of moments (GMM) approach to produce unbiased short- and long-run estimates of economies of scale, using the number of contacts made by HIV counsellors for HB-HCT as the scale variable. We also estimated the mediating effect of the contact quality – measured as the proportion of HIV tests performed among all contacts eligible for an HIV test – on scale economies. The mean cost (standard deviation) of universal and repeated HB-HCT was $24.2 (13.7) per contact, $1694.3 (1527.8) per new HIV diagnosis, and $269.2 (279.0) per appropriate referral to HIV care. The GMM estimations revealed the presence of economies of scale, with a 1% increase in the number of contacts for HB-HCT leading to a 0.27% decrease in the mean cost. Our results also suggested a significant long-run relationship between mean cost and scale, with a 1% increase in the scale leading to a 0.36% decrease in mean cost in the long run. Overall, we showed that significant cost savings can be made from increasing population coverage. Nevertheless, there is a risk that this gain is made at the expense of quality: the higher the quality of HB-HCT activities, the lower the economies of scale.

## Highlights

• We estimated the costs of repeated home-based HIV counselling and testing (HB-HCT).
• The mean cost per new HIV diagnosis, which increased over HB-HCT rounds, was $1694.
• However, the mean cost per appropriate referral to HIV care was $269.
• A 1% increase in the scale of HB-HCT reduced the average cost per contact by 0.27%.
• Expanding the population coverage of HB-HCT offers opportunities for cost savings.

## 1 Introduction

In sub-Saharan Africa, the proportion of people living with HIV (PLHIV) aware of their HIV status increased from 53% in 2010 to 84% in 2020 (Giguère et al., 2021). Despite significant progress, increasing HIV testing coverage remains a major challenge to achieving universal access to HIV prevention, treatment and care in the region and, in turn, to reducing HIV incidence. Universal test and treat (UTT) (i.e., wide-ranging universal testing combined with HIV treatment offered immediately to all people tested HIV-positive irrespective of CD4 count), is a key strategy to reach that goal (World Health Organization, 2015a, 2016). More specifically, in concentrated and generalised HIV epidemic settings, the WHO recommends offering community-based HIV testing services, including home-based testing, in addition to provider-initiated testing and counselling services (World Health Organization, 2015b).

The following four large-scale UTT community-randomised trials were recently conducted to assess whether adopting a UTT strategy might achieve the HIV elimination targets and lead to a marked decrease in community HIV incidence: ANRS 12249/Treatment as Prevention (TasP) in South Africa (Iwuji et al., 2018), BCPP/YaTsie in Botswana (Makhema et al., 2019), HPTN 071/PopART in Zambia and South Africa (Hayes et al., 2019), and SEARCH in Kenya and Uganda (Havlir et al., 2019). Current evidence suggests that the UTT strategy can lead to high population-level viral suppression and lower HIV incidence when associated with effective linkage to HIV care, rapid antiretroviral therapy (ART) initiation, and patient-centred care (Havlir et al., 2020).

With regard to the testing component of UTT, the four trials mentioned above successfully achieved high HIV testing coverage and HIV status awareness, using a strategy of home-based HIV counselling and testing (HB-HCT) combined with provider-initiated counselling and testing (Havlir et al., 2020). Furthermore, several studies have found that HB-HCT is a feasible and effective strategy and can significantly increase HIV testing uptake (Moshoeu et al., 2019). However, testing raises important cost-efficiency and sustainability questions. To stop HIV transmission, UTT must identify newly HIV-infected individuals quickly and initiate treatment immediately. This necessitates regular and repeated HIV testing. Repeated HB-HCT was described as more acceptable than clinic-based testing (Orne-Gliemann et al., 2016). On the one hand, while repeated HB-HCT may be effective in increasing HIV testing coverage, it may also lead to high costs, since the positivity rate (i.e., the number of individuals tested HIV-positive among all HIV-tested individuals) is likely to decrease with the number of HB-HCT campaigns or rounds. As a result, the mean cost per individual tested HIV-positive is likely to increase together with the mean cost per individual tested. On the other hand, the scaling up of HB-HCT may lead to a lower mean cost per individual tested if economies of scale are present, and therefore to efficiency gains. We define scaling up here as increasing the coverage of health interventions (i.e., extending their geographical reach such that they benefit a greater number of people) in order to support policy and programme development at a large or national scale (Mangham and Hanson, 2010; Simmons et al., 2007).

Existing costing studies are mainly based on HB-HCT campaigns with single testing which are conducted over a relatively short time (Hauck, 2019). By its very definition, UTT ultimately must be implemented at the national level. However, there is currently little data on the costs of repeated HB-HCT as part of a UTT strategy (Thomas et al., 2021), and no evidence of the existence of economies of scale (i.e., decreasing mean costs when the scale increases) to inform decisions about the scaling up of HB-HCT activities.

In a context of constrained resources, limited HIV budgets, and competing priorities, such as non-communicable disease control programmes, public decision-makers are likely to benefit from knowing not only what the costs of universal and repeated HB-HCT are, but also the evolution of these costs when the scale increases (i.e., when population coverage expands). As suggested by two studies in India (Lépine et al., 2015, 2016), the scale-up of the intervention may be an important driver of costs of HIV prevention activities, especially when there are large fixed costs or learning-by-doing effects. The presence of scale economies implies that cost savings may be obtained from scaling up complex interventions such as HB-HCT. Although this is critical information to guide decisions about universal testing implementation, there is currently no evidence of the (causal) effect that the scaling up of HB-HCT activities has on the mean cost of HB-HCT. Measuring and accounting for the quality of UTT activities is also needed (Nachega et al., 2021), especially when activities are conducted among hard-to-reach populations.

Based on primary data collected in the ANRS 12249 TasP trial, implemented sequentially over the period 2012–2016 in South Africa, the present study aimed to estimate the costs of universal and repeated HB-HCT when implemented on a large scale, as part of a UTT strategy, and to assess the presence of potential economies (or diseconomies) of scale when increasing population coverage of the HB-HCT intervention.

## 2 Methods

### 2.1 Trial design and procedures

ANRS 12249 TasP was a phased two-arm cluster-randomised trial conducted by the Africa Health Research Institute (AHRI) between March 2012 and June 2016 in the Hlabisa sub-district, northern KwaZulu-Natal, South Africa (see Iwuji et al. (2013) and Orne-Gliemann et al. (2015) for a full description of the protocol). The study area is mainly rural, with a population of approximately 28,000 isiZulu-speaking eligible resident adults (≥16 years) and a 30% HIV prevalence in adults at baseline (Iwuji et al., 2016).

The main objective of the trial was to assess whether universal HIV testing of all the adult population, followed by referral of persons tested HIV-positive to clinics for immediate ART initiation, would reduce HIV incidence in the area. The trial comprised 22 clusters designed to cover an average population of approximately 1000 resident adults per cluster and stratified based on adult HIV prevalence. Clusters were randomly allocated (1:1) to either the control or intervention arm. The UTT strategy had two main components: (i) repeated home-based HIV testing implemented in all clusters (i.e., irrespective of the randomisation group), and (ii) ART initiation offered either immediately irrespective of the CD4 cell count in the intervention clusters, or according to national guidelines (initially starting at CD4 counts ≤350 cells per μL and then <500 cells per μL from January 2015) in the control clusters.

The present study focused on the universal HB-HCT component of the ANRS 12249 TasP trial, which was implemented in the 22 clusters between March 2012 and April 2016 using a three-phased approach: four clusters opened in March 2012, six additional clusters opened in January 2013 and twelve more in June 2014. In each cluster, HB-HCT was offered every six to eight months to all eligible household members (i.e., residents 16 years or older). During the trial period, seven testing rounds were implemented in the first four clusters, six in the six subsequent clusters, and four in the last twelve clusters.

Before the first round of HB-HCT in each cluster, roadshows were organised to inform the community about the trial and HB-HCT activities. Subsequently, HIV counsellors visited all households in the cluster and registered all adults ≥16 years old residing in each household (initial census), with the help of the household head (or another adult household member if the head was absent). All eligible adults present in the household at the time of the visit were contacted and offered HB-HCT. After obtaining written informed consent, HIV counsellors conducted individual pre-test counselling interviews, performed point-of-care (POC) rapid HIV tests (except for participants who self-reported being HIV-positive), and delivered test results with post-test counselling. Irrespective of agreeing or not to be tested for HIV, all individuals were invited to provide a dried blood spot (DBS) for research purposes (incidence measurement). Individuals identified as HIV-positive through DBS but who refused an HIV rapid test during a given HB-HCT round were not notified of their DBS result, and were re-invited to test for HIV in a subsequent round. All participants identified as HIV-positive, whether through rapid HIV testing or self-report, were referred for ART initiation to a trial clinic dedicated to their cluster, usually located less than 5 km or a 45-min walking distance from their home.

The ANRS 12249 TasP trial showed that an average of 91.5% of HIV-positive participants were aware of their HIV status by the end of the trial. Of these, 58.0% were on ART, and 85.3% of the latter achieved viral suppression (Iwuji et al., 2018). However, no differences were found between the intervention arm where immediate ART initiation was implemented and the control arm where ART initiation was based on national guidelines. Larmarange et al. (2019) showed that most of the gains in the HIV care cascade were due to strategies to maximise testing and linkage to care – which were implemented in both arms – with universal testing being the main driver of improvements.

### 2.2 Outcomes and cost measurements over the trial period

The main data source for the present analysis was the ANRS 12249 TasP trial database, which provided information on trial registrations and trial exits, on the uptake and results of home-based rapid HIV testing, and on clinic visits and biological data of PLHIV seen in trial clinics. Two additional data sources, matched with the trial database, captured information from PLHIV seen in local governmental clinics and were described in detail by Larmarange et al. (2019).

The following eight outcomes were measured for each cluster and each month (cluster-month) from March 2012 to April 2016: (a) the number of residents aged ≥16 years registered in the trial (i.e., the target population eligible for HB-HCT, whose enumeration was updated each survey round to account for in- and out-migration, individuals turning 16 years of age, and deaths); (b) the number of contacts made by HIV counsellors to offer HB-HCT; (c) the number of contacts eligible for an HIV test according to trial procedures (i.e., all contacts except those who self-reported being HIV-positive to the field worker); (d) the number of rapid HIV tests performed; (e) the number of positive rapid HIV tests; and (f) the number of new HIV diagnoses (i.e., contacts newly diagnosed as HIV positive, taking into account previous contacts and records in local governmental clinics). We also considered an outcome relating HIV testing to linkage to care: (g) the number of appropriate referrals to HIV care (i.e., contacts where the person was ascertained HIV positive through rapid testing or self-report, and was not currently in HIV care in a local governmental clinic or a trial clinic).

Costs were assessed from a provider perspective using a top-down micro-costing methodology with data collected prospectively during the trial period. These data were complemented by relevant national sources, programme records, financial and activity reports, as well as interviews with the staff involved in the trial implementation at the AHRI. Costs included recurrent costs (personnel, transport, communication, and HIV tests and supplies), and capital costs (vehicles and other capital assets such as mobile phones, GPS receivers and backpacks). Data on salaries and recurrent transport costs were directly obtained from the programme financial reports. In the present analysis, we only considered the share of wages corresponding to the work time devoted to HB-HCT activities. This was estimated based on staff interviews. The majority of the staff involved in HB-HCT activities devoted their whole time to these activities, and therefore 100% of their wages were allocated to HB-HCT, except for the trial coordinator and the manager in charge of the communication activities in the community, for whom only 30% and 10% of the time were allocated to HB-HCT. The market value of resources (prices and quantities) used for the roadshows (communication activities) was evaluated in 2016 based on interviews with the manager in charge of these activities. Unit costs of HIV POC rapid tests and related supplies were taken from the relevant national source (National Health Laboratory Service, 2013). Prices and quantities of capital assets including vehicles, mobile phones, GPS receivers and backpacks were calculated from programme records and financial reports. The monthly economic value of capital assets was computed based on their acquisition date and expected useful life (3 or 5 years).

The following procedure was used to aggregate costs and adjust them to take into account inflation and discounting over the study period. We first calculated the total monthly costs (in South African rands) for each month of the study period, as the sum of all monthly recurrent and capital costs devoted to HB-HCT activities. These monthly costs were converted to United States dollars ($) using year-specific exchange rates (Board of Governors of the Federal Reserve System (US), 2020) and then converted from nominal to real values (expressed in $2016) using the annual US GDP deflator (Organization for Economic Co-operation and Development, 2020). We took the present value of this cost stream using a monthly discount rate of 0.25% (corresponding to a 3% annual discount rate, the base month being March 2012). Costs per cluster-month in $2016 were obtained by allocating the total monthly costs (inflation-adjusted and discounted) to each cluster-month based on the share of the population reached (i.e., the number of eligible adult residents contacted by an HIV counsellor for HB-HCT in the corresponding month relative to the total number of individuals contacted for HB-HCT in all clusters). Finally, we computed the unit costs of the intervention per cluster-month in $2016 for each outcome as the mean cost per contact, per contact eligible for an HIV test, per rapid HIV test, and per new positive HIV test.

### 2.3 Methodology for estimating economies of scale

Using the longitudinal cost data which we estimated per cluster-month over the period from March 2012 to June 2016, we implemented a series of panel regression models to obtain unbiased estimates of economies of scale in HB-HCT activities. The scale of HB-HCT was measured as the number of contacts made by HIV counsellors, corresponding to the population reached by the intervention (i.e., offered HB-HCT).

#### 2.3.1 Fixed-effects panel regression models

We first used the standard fixed-effect estimator, which is usually considered as a starting point in the estimation of economies of scale (for instance in Lépine et al. (2015)). Accordingly, we estimated the following fixed-effects panel regression model of the average cost (AC) of HB-HCT cluster i(i=1,…,n) in cluster-specific month-year t(t=1,…,T):

(1) $$Log{\left(AC\right)}_{it}=\beta Log{\left(Scale\right)}_{it}+\omega_{t}+\mu_{i}+\varepsilon_{it}$$

where $Log{\left(Scale\right)}_{it}$ is the log of the number of contacts for HB-HCT (i.e., the scale of HB-HCT activities) in cluster and month-year t, $\omega_{t}$ are month-year dummies, $\mu_{i}$ are HB-HCT cluster fixed effects, and $\varepsilon_{it}$ is an idiosyncratic error term. We chose a log transformation of the (right-skewed) scale variable rather than a quadratic transformation, as it explained a larger fraction of the variance. The coefficient of interest is β, measuring the effect of a 1% increase in the scale of HB-HCT activities on the average cost of HB-HCT activities.

By considering cluster-specific time within the trial (month-year since the opening of each cluster) rather than calendar time (month-year since the beginning of the trial), the model accounts for the sequential implementation of HB-HCT activities. Time fixed effects (cluster-specific month-year) were included in the model to account for the potential effects of unexpected variations or special events on the outcome variable, including the effect of the repeated HB-HCT strategy (i.e., implemented over successive rounds) on average costs. Standard errors, clustered by HB-HCT cluster, were therefore robust to cross-sectional heteroskedasticity and within-panel autocorrelation.

By applying a within transformation, Model 1 accounts for HB-HCT cluster-level unobserved heterogeneity. However, this model might be misspecified if there is serial correlation in the error process arising from persistence in the average cost series.

#### 2.3.2 Dynamic panel models

To avoid possible misspecification bias, we estimated a dynamic panel data model (containing a lagged dependent variable), allowing for a partial adjustment mechanism. Moreover, the lagged dependent variable is likely to work as a proxy for omitted variables (Wooldridge, 2010). However, dynamic panel data models estimated by a standard fixed-effects estimator are likely to suffer from the so-called Nickell bias, arising from the correlation between the fixed effects and the regressors owing to the lags of the dependent variable, especially when the number of time periods is relatively small (Nickell, 1981).

For this reason, we used the generalised method of moments (GMM) to estimate the causal effect of scaling up HB-HCT activities on the average cost and thereby obtain unbiased estimations of economies of scale. More specifically, we used the two-step system GMM developed by Blundell and Bond (1998):

(2) $$Log{\left(AC\right)}_{it}=\alpha Log{\left(AC\right)}_{i,t-1}+\beta Log{\left(Scale\right)}_{it}+\omega_{t}+\mu_{i}+\varepsilon_{it}$$

where $E\left[\mu_{i}\right]=E\left[\varepsilon_{it}\right]=E\left[\mu_{i}*\varepsilon_{it}\right]=0$.

Note that the long-run estimate of economies of scale can be defined as β/(1−α). We applied the finite-sample correction of the two-step covariance matrix proposed by Windmeijer (2005), which has been shown to make two-step robust estimations more efficient than one-step robust estimations, especially for system GMM (Roodman, 2009a). We also used the forward orthogonal deviations proposed by Arellano and Bover (1995) instead of first-differencing, as the latter transformation magnifies gaps in unbalanced panels (Roodman, 2009a). Cluster-specific year dummies were included in the model instead of cluster-specific month-year dummies to avoid the common issue of instrument proliferation (Roodman, 2009b).

All explanatory variables were treated as endogenous or predetermined, except time dummies which were treated as exogenous following Roodman (2009a). Time dummies were considered as instruments in the levels equation only. The scale variable was instrumented using its own first to third lags in the transformed (orthogonal deviations) equation and its contemporaneous first difference in the levels equation. The lagged dependent variable was instrumented using its first and second lags in the transformed equation and its first difference in the levels equation. All predetermined or endogenous instruments were collapsed to limit the instrument count (Roodman, 2009a).

#### 2.3.3 Dynamic panel models including the quality of HB-HCT activities

Finally, two additional models were estimated to analyse further the relationship between the scale and quality (in the economic sense) of HB-HCT activities and the average cost. Model 3 consists of Model 2 with the addition of the proportion of HIV tests performed among all contacts eligible for an HIV test (i.e., all contacts except those who self-reported being HIV-positive to the field worker) per cluster-month. This variable represents a better proxy for contact quality than the proportion of HIV tests performed among all contacts, since the latter is likely to be influenced by the prevalent infections identified in previous HB-HCT rounds. In the models, this variable was treated as predetermined and instrumented using its first lag.

To test whether the effect of the scale on the mean cost depended on the quality of the HB-HCT, Model 4 further included an interaction term between the scale and contact quality. A significant negative interaction term would indicate the existence of a trade-off between the quality and the scale of the activities, in the sense that an increase in the quality of HB-HCT activities would be associated with potentially lower economies of scale.

The following diagnostic tests were performed in all GMM estimations. First, we tested for the presence of second-order serial correlation in the first-differenced residuals using the Arellano-Bond AR(2) test, under the null hypothesis of no serial correlation (Arellano and Bond, 1991). Second, we used the Hansen (1982) J test statistic for over-identifying restrictions to assess the joint validity of the instruments, as the Sargan (1958) test is not relevant when using the two-step system GMM estimator (Roodman, 2009a). Note that, in Models 3 and 4, the Hansen test is likely to be weakened by the increase in instrument count implied by the inclusion of the contact quality variable.

All analyses were performed using Stata (StataCorp, 2019). The two-step system GMM estimation was performed using the *xtabond2* Stata module developed by Roodman (2009a).

## 3 Results

### 3.1 Outcomes and costs

**Fig. 1:**
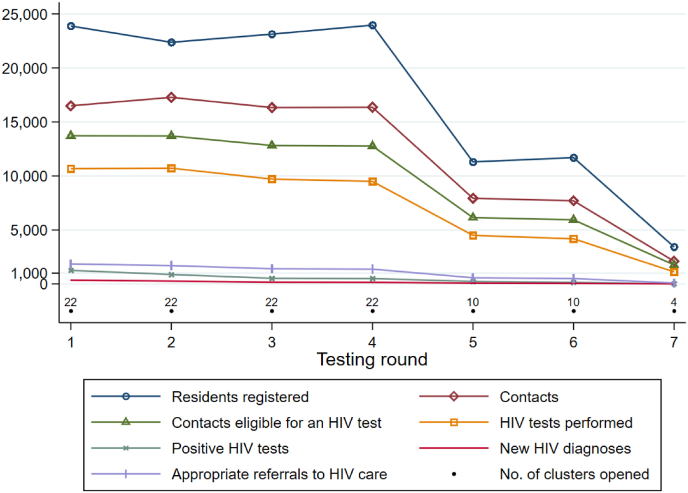
Total number of residents aged ≥16 years registered, of contacts made for HB-HCT, of contacts eligible for an HIV test, of HIV tests performed, of positive HIV tests, of new HIV diagnoses, and of appropriate referrals to HIV care, per testing round.

As the trial implemented universal and repeated HB-HCT, each eligible individual was likely to be contacted and/or tested several times during the trial period. Accordingly, we present all HB-HCT outcomes and costs per testing round. Outcomes per testing round are depicted in Fig. 1. Table 1 provides detailed information on the outcomes and costs of HB-HCT both per testing round and over the whole study period (March 2012–April 2016). Among the 28,347 eligible adult residents registered at least once over the study period, 81,611 distinct contacts were made. Of these, 64,804 were eligible for an HIV test. Of the 49,046 HIV tests performed, 3510 were positive. 1046 new HIV diagnoses were made, and 7274 contacts were referred to HIV care.

**Table 1:** Outcomes and costs (in US$ 2016) of home-based HIV counselling and testing, per testing round.

	**Round 1**	**Round 2**	**Round 3**	**Round 4**	**Round 5**	**Round 6**	**Round 7**	**Whole period (March 2012–April 2016)**
**Clusters opened**	22	22	22	22	10	10	4	22
**(a) No. of individuals registered**	21,419	21,312	21,902	22,117	10,808	10,422	2918	28,347
**(b) No. of contacts for HB-HCT**	16,575	17,108	16,245	14,041	7982	7555	2105	81,611
**(c) No. of contacts eligible for an HIV test**	13,652	13,696	12,750	10,939	6186	5820	1761	64,804
**(d) No. of HIV tests performed**	10,627	10,713	9666	8263	4533	4127	1117	49,046
**(e) No. of positive HIV tests**	1254	873	523	477	231	134	18	3510
**(f) No. of new HIV diagnoses**	356	263	163	134	74	52	4	1046
**(g) No. of appropriate referrals to HIV care**	1846	1696	1401	1185	575	486	85	7274
**Total cost**	526,226.3	433,370.0	312,313.5	312,035.3	166,285.3	159,022.0	69,071.2	1,978,323.6
**Cost per contact**								
Mean	32.0	25.1	19.2	22.2	20.8	21.0	32.8	24.2
(SD)	(21.3)	(13.0)	(4.9)	(6.6)	(15.2)	(5.4)	(7.7)	(13.7)
Median	21.4	19.7	19.6	19.9	15.4	19.1	36.8	19.8
(IQR)	(19.8–30.5)	(18.0–26.0)	(14.8–23.4)	(18.7–22.1)	(14.2–19.7)	(18.8–19.8)	(36.6–37.1)	(17.9–23.7)
**Cost per contact eligible for an HIV test**								
Mean	38.5	31.6	24.5	28.5	26.9	27.3	39.2	30.5
(SD)	(23.1)	(15.2)	(5.8)	(7.1)	(18.3)	(6.5)	(9.0)	(15.4)
Median	27.8	25.0	24.6	26.5	20.7	26.4	43.7	25.4
(IQR)	(24.7–44.4)	(22.4–35.1)	(19.4–29.4)	(24.2–29.9)	(18.6–23.2)	(23.2–28.3)	(43.3–44.3)	(22.4–31.3)
**Cost per HIV test performed**								
Mean	49.5	40.4	32.3	37.7	36.5	38.5	61.8	40.3
(SD)	(30.8)	(20.7)	(8.6)	(9.4)	(23.2)	(8.8)	(15.8)	(20.7)
Median	36.2	31.3	31.3	34.9	28.5	36.8	64.7	34.6
(IQR)	(30.1–62.9)	(28.4–45.7)	(25.2–37.0)	(31.8–38.3)	(26.3–31.0)	(34.4–40.3)	(63.5–70.5)	(28.8–40.5)
**Cost per positive HIV test**								
Mean	412.0	489.7	586.9	614.1	691.3	1130.8	3579.6	546.9
(SD)	(345.2)	(413.5)	(443.9)	(483.8)	(640.7)	(825.6)	(5159.2)	(620.2)
Median	264.6	363.2	496.3	463.1	643.8	916.0	2433.6	376.0
(IQR)	(228.2–466.0)	(317.9–454.1)	(281.8–621.0)	(372.8–637.8)	(341.2–667.3)	(563.9–1532.6)	(1140.8–3645.4)	(275.5–621.0)
**Cost per new HIV diagnosis**								
Mean	1430.7	1513.8	1698.8	2039.5	2122.7	2405.6	8110.3	1694.3
(SD)	(1176.1)	(1103.9)	(1444.2)	(1386.6)	(2398.1)	(2046.0)	(8426.0)	(1527.8)
Median	1041.4	1201.9	1505.4	1594.7	1319.6	1374.0	6323.7	1207.6
(IQR)	(744.8–1531.0)	(888.2–1710.3)	(976.9–1737.0)	(1173.0–2383.8)	(764.7–2446.7)	(1179.1–3041.2)	(1711.2–14,509.3)	(914.9–1906.7)
**Cost per appropriate referral to HIV care**								
Mean	280.8	254.2	221.5	261.8	281.4	323.7	811.0	269.2
(SD)	(358.7)	(293.4)	(124.5)	(158.6)	(341.3)	(213.5)	(499.9)	(279.0)
Median	170.7	174.8	205.5	213.3	183.9	251.3	912.6	185.2
(IQR)	(136.5–242.7)	(140.5–237.3)	(137.0–259.3)	(166.4–291.6)	(160.5–247.4)	(220.2–371.7)	(322.8–1063.7)	(150.8–273.6)

Notes: Monetary amounts are provided in US$ (year 2016 values).

Definitions: (a) number of residents aged ≥16 years registered in the ANRS 12249 TasP trial (i.e., the target population eligible for HB-HCT, whose enumeration was updated at each survey round to account for in- and out-migration, individuals turning 16, and deaths), (b) number of contacts made by HIV counsellors to offer HB-HCT, (c) number of contacts eligible for an HIV test according to trial procedures (i.e., all contacts except those who self-reported being HIV-positive to the field worker), (d) number of rapid HIV tests performed, (e) number of positive rapid HIV tests, (f) number of new HIV diagnoses (contacts newly diagnosed as HIV positive, taking into account previous contacts and records in local governmental clinics), and (g) number of appropriate referrals to HIV care (i.e., contacts where the person was ascertained HIV positive through rapid testing or self-report, and was not currently in HIV care in a local governmental clinic or a trial clinic).

Abbreviations: HB-HCT = home-based HIV counselling and testing. SD = standard deviation. IQR = interquartile range.

The total cost devoted to HB-HCT activities over the study period amounted to $1,978,324. The mean costs (SD) per outcomes were as follows: $24.2 (13.7) per contact, $30.5 (15.4) per contact eligible for an HIV test, $40.3 (20.7) per HIV test, $546.9 (620.2) per positive HIV test, $1694.3 (1527.8) per new HIV diagnosis, and $269.2 (279.0) per appropriate referral to HIV care.

Table 2 provides a cost breakdown of HB-HCT per testing round and for the whole study period. Personnel costs represented the highest cost category (57.9% of total costs), followed by the cost of capital (18.0%), transport (11.8%), HIV test and supplies (9.8%), and lastly communication (2.5%).

**Table 2:** Cost breakdown per testing round.

	**Round 1**	**Round 2**	**Round 3**	**Round 4**	**Round 5**	**Round 6**	**Round 7**	**Whole period**
**Recurrent costs**	430,752.8	337,962.9	261,591.0	264,798.9	135,940.3	133,588.4	57,399.0	1,622,033.3
(% of total costs)	(81.9)	(78.0)	(83.8)	(84.9)	(81.8)	(84.0)	(83.1)	(82.0)
**Personnel**	288,516.0	230,595.4	181,635.0	194,089.5	110,820.5	95,941.6	43,586.4	1,145,184.3
(% of total costs)	(54.8)	(53.2)	(58.2)	(62.2)	(66.6)	(60.3)	(63.1)	(57.9)
**Transport**	85,959.8	52,750.2	32,452.8	31,322.6	3736.8	18,687.6	8476.5	233,386.3
(% of total costs)	(16.3)	(12.2)	(10.4)	(10.0)	(2.2)	(11.8)	(12.3)	(11.8)
**Communication**	13,169.7	11,894.6	9527.4	7276.3	3777.0	3168.5	1073.7	49,887.2
(% of total costs)	(2.5)	(2.7)	(3.1)	(2.3)	(2.3)	(2.0)	(1.6)	(2.5)
**HIV tests and supplies**	43,107.3	42,722.8	37,975.7	32,110.6	17,606.1	15,790.6	4262.4	193,575.5
(% of total costs)	(8.2)	(9.9)	(12.2)	(10.3)	(10.6)	(9.9)	(6.2)	(9.8)
**Capital costs**	95,473.5	95,407.0	50,722.6	47,236.4	30,345.0	25,433.7	11,672.2	356,290.4
(% of total costs)	(18.1)	(22.0)	(16.2)	(15.1)	(18.2)	(16.0)	(16.9)	(18.0)
**Total costs**	526,226.3	433,370.0	312,313.5	312,035.3	166,285.3	159,022.0	69,071.2	1,978,323.6

Notes: Monetary amounts are provided in US$ (year 2016 values).

Outcomes and costs over calendar time are provided in the Electronic Supplementary Material. Figure A1 depicts the outcomes per month over the trial period. Outcomes and costs of HB-HCT per year are provided in Table A1, and a yearly cost breakdown of HB-HCT is presented in Table A2.

### 3.2 Estimation of economies of scale

**Fig. 2:**
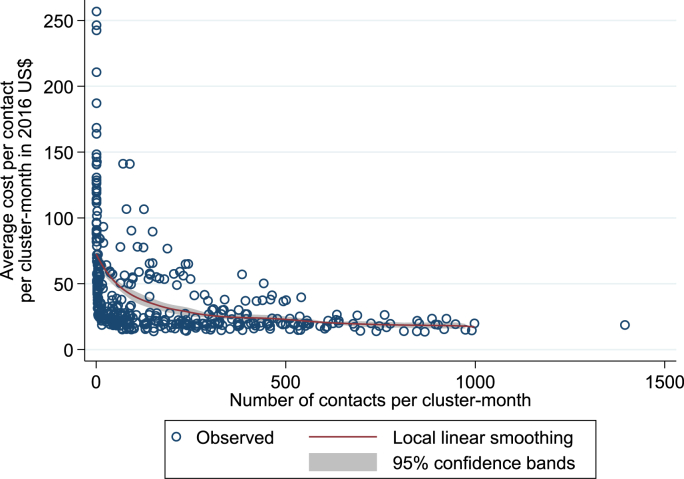
Relationship between the average cost and the number of contacts per cluster-month. **Legend:** The regression is based on a quartic (biweight) kernel function and a bandwidth of 200. Note that, based on the regression of smoothed on original values of the average cost for the values of the number of contacts, the R-squared and root mean squared error statistics were equal to 0.311 and 30.8, respectively.

The relationship between the average cost and the scale of the activities (i.e., the number of contacts for HB-HCT) is depicted in Fig. 2. More specifically, Fig. 2 shows the regression curve of the average cost as a function of the number of contacts per cluster-month – estimated using kernel-weighted local linear smoothing – together with the scatter plot of observed values. It highlights a negative relationship between the scale and the cost of HB-HCT activities and suggests that diseconomies of scale (i.e., an increase in the average cost at high volumes of activity) were unlikely to arise.

Table 3 provides the descriptive statistics of the variables used in the models computed per cluster-month (391 observations), and all regression results (Model 1 to Model 4) are presented in Table 4.

**Table 3:** Descriptive statistics of the variables per cluster-month.

**Variable**	**Obs.**	**Mean**	**Standard deviation**	**Median**	**Interquartile range**	**Min**	**Max**
**Average cost per contact per cluster-month**	391	41.5	37.1	25.8	19.6–53.4	13.5	256.8
**Log(Average cost per contact per cluster-month)**	391	3.5	0.7	3.3	3.0–4.0	2.6	5.5
**Scale (number of contacts)**	391	208.7	244.5	97	12–330	1	1395
**Log(Scale)**	391	4.1	2.1	4.6	2.5–5.8	0	7.2
**Number of HIV tests performed**	391	125.4	144.5	62	7–206	0	821
**Contact quality (proportion of HIV tests performed among all contacts eligible for an HIV test)**	382	0.728	0.187	0.749	0.667–0.831	0	1

Notes: Monetary amounts are provided in US$ (year 2016 values).

**Table 4:** Estimation of economies of scale: regression results.

	**Model 1 Fixed-effects**	**Model 2 System GMM**	**Model 3 System GMM**	**Model 4 System GMM**
**Log(AC)**_**i,t-1**_		0.250** (0.072)	0.230** (0.075)	0.171* (0.064)
**Log(Scale)**	−0.239*** (0.016)	−0.268*** (0.014)	−0.246*** (0.029)	−0.601** (0.192)
**Contact quality**			0.241 (0.226)	−0.544 (0.500)
**Log(Scale) x Contact quality**				0.482^+^ (0.260)
**Constant**	4.879*** (0.108)	3.772*** (0.266)	3.575*** (0.250)	4.318*** (0.449)
**Time fixed effects**	Yes	Yes	Yes	Yes
**HB-HCT clusters fixed effects**	Yes	Yes	Yes	Yes
**R-squared**	0.657			
**No. of groups**	22	22	22	22
**No. of time periods per group (average)**	17.8	14.1	13.7	13.7
**No. of observations**	391	310	302	302
**No. of instruments**		11	13	13
**Arellano-Bond test for AR(2) (p-value)**		0.425	0.749	0.768
**Hansen test of over-identification (p-value)**		0.105	0.145	0.569

Notes:+p < 0.1, ∗ p < 0.05, ∗∗ p < 0.01, ∗∗∗ p < 0.001. Standard errors in parentheses (cluster-robust in Model (1), and Windmeijer-corrected cluster-robust in Model (2)).

The results of Model 1 – which estimated economies of scale using the standard fixed-effect estimator – show that a 1% increase in the HB-HCT scale led to a 0.24% decrease in the average cost of HB-HCT activities. However, as discussed in the methodology subsection, this estimate of scale economies may be biased if there is serial correlation in the error process. Such a bias was confirmed when estimating Model 2 (two-step system GMM estimation of a dynamic panel model). The Arellano-Bond test for AR(2) indicated that we cannot reject the null hypothesis of no second-order serial correlation (p = 0.425), implying that the system GMM estimator was consistent. In addition, the Hansen test did not reject the null hypothesis that all over-identifying restrictions were jointly valid (p = 0.105). The instrument count (11) was not too large with respect to the number of HB-HCT clusters (22), thus avoiding the problem of instrument proliferation.

The results of Model 2 show that the unbiased estimate of economies of scale was equal to −0.268, indicating that a 1% increase in the scale of HB-HCT activities reduced the average cost by 0.27%. Our estimate of the lagged dependent variable (i.e., 0.250, p < 0.01) is consistent since (i) it is dynamically stable (below unity), and (ii) it falls within the range whose lower bound is given by the downward-biased estimate of the lagged dependent variable estimated by the standard fixed-effects estimator (i.e., 0.237, p < 0.001) and whose upper bound is given by the upward-biased estimate of the lagged dependent variable estimated by the ordinary least-squares estimator (i.e.,0.250, p < 0.001) (Roodman, 2009a).

The results of Model 2 also indicate a significant long-run relationship between the average cost and the scale: the long-run estimate of economies of scale (calculated as β/(1−α)) is equal to −0.357 (p < 0.001), indicating that a 1% increase in the scale is associated with a 0.36% decrease in the average cost in the long run, all other things being equal. Accordingly, the long-run effect (−0.357) is larger than the short-run effect (−0.268).

**Fig. 3:**
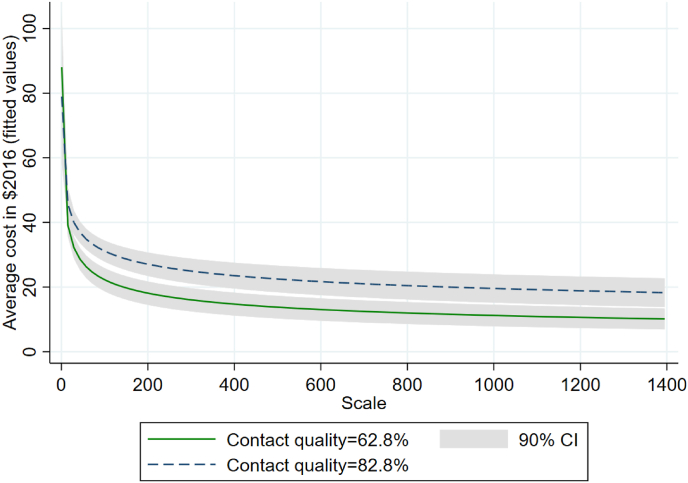
Effect of scaling up HB-HCT activities on the average cost at two different values of contact quality (fitted values estimated in Model 4). **Legend:** The figure plots the fitted values of average cost (with 90% confidence intervals, CI) estimated in Model 4 across the range of the scale variable at two different values of contact quality (62.8% and 82.8%). As the mean contact quality was 0.728 (i.e., on average 72.8% of all contacts eligible for an HIV test were eventually tested for HIV), the two values considered are 10 percentage points below and above the sample average. For instance, for a contact quality of 62.8%, increasing the scale from 100 to 200 contacts in a cluster-month would decrease the average cost per contact from $21.9 to $17.8 (p < 0.001). On the other hand, the average cost would decrease from $30.6 to $26.6 (p < 0.001) for the same increase in scale but a contact quality of 82.8%. Although both increases in the number of contacts would lead to significant economies of scale, the decrease in the average cost would be lower for a higher contact quality (−13.1% versus −18.7% when 62.8% of the contacts eligible for an HIV test were eventually tested, p < 0.10).

Although the results of Model 3 show that the variable proxying the quality of HB-HCT activities alone did not have a significant effect on the average cost, the negative interaction term (p < 0.10) in Model 4 indicates that the higher the contact quality, the lower the economies of scale. This is also illustrated in Fig. 3, which plots the fitted values of average costs estimated in Model 4 (with 90% confidence intervals) across the range of the scale variable. These fitted values are plotted for two different values of contact quality: 62.8% and 82.8%, corresponding to 10 percentage points below and above the sample average, respectively, since on average 72.8% of the contacts eligible for an HIV test were eventually tested for HIV (as shown in Table 3). Fig. 3 shows that economies of scale were less pronounced for higher proportions of HIV tests performed among all contacts eligible for an HIV test. The estimated average marginal effects of the scale on the average cost were −0.298 (p < 0.001) and −0.202 (p < 0.001) for a contact quality of 0.628 and 0.828, respectively. These findings indicate that a 1% increase in the scale led to a 0.30% decrease in the average cost for a contact quality of 62.8%, while the same percentage increase in the scale led to a 0.20% decrease in the average cost when 82.8% of the contacts eligible for an HIV test were eventually tested. Although scaling up HB-HCT activities led to significant economies of scale in both cases, the decrease in the average cost was lower for higher contact quality.

## 4 Discussion

We assessed the costs of implementing large-scale, universal, and repeated HB-HCT as part of the UTT strategy implemented in the ANRS 12249 TasP trial between 2012 and 2016 in rural South Africa. The longitudinal nature of the trial data also allowed us to provide the first evidence, to our knowledge, of the presence of economies of scale when increasing the population coverage of HB-HCT.

### 4.1 The cost of repeated HB-HCT

Overall, the mean costs estimated in our study are higher than those reported by Hauck (2019), who conducted a comparative review of existing costing studies of HB-HCT in sub-Saharan Africa. More specifically, the review estimated an average cost per person tested for HIV of $22.8 (SD 14.5) across 14 studies (with large variations in cost estimates), compared with a cost per HIV test of $40.3 (SD 20.7) in TasP. Furthermore, it found an average cost per individual tested HIV-positive of $439.4 (SD 399.7) across 12 studies, compared with a cost per positive HIV test of $546.9 (SD 620.2) in TasP.

These differences between TasP and previous HB-HCT estimates are partly explained by the fact that TasP implemented six-monthly repeated HIV testing, while the studies reviewed by Hauck (2019) implemented single testing and were generally conducted over a relatively short duration. Within a repeated testing strategy, the first round of HB-HCT is likely to identify prevalent infections, while subsequent rounds are likely to identify incident infections. In such a setting, the HIV positivity rate is expected to decrease over successive rounds, leading to a likely increase in the cost per positive HIV test over successive rounds, as can be seen in Table 1. It would therefore appear relevant to compare the estimates reported in Hauck (2019) with those for the first round of HB-HCT in TasP. In TasP, the mean cost per positive HIV test was $412.0 (SD 345.2) for the first round of HB-HCT. That is slightly lower than the $439.4 (SD 399.7) found in Hauck (2019).

Costing studies of HIV testing often rely mainly on the cost per new HIV diagnosis, which was relatively high in TasP (i.e., $1694.3, SD 1527.8). The latter result is not surprising given the high proportion of PLHIV already aware of their HIV status in the study area (i.e., the ‘first 95’ of the HIV care cascade as defined in the 2030 Joint United Nations Programme on HIV/AIDS (UNAIDS) 95-95-95 targets), estimated at 80% at baseline (Larmarange et al., 2019).

However, it would be reductive to consider diagnoses as the sole gain of HIV testing, since the ultimate goal of UTT approaches is to achieve viral suppression at the population level. The last WHO guidelines on HIV prevention, testing, treatment, service delivery and monitoring, underline that further research is needed on strategies to support effective linkage to HIV services among PLHIV already aware of their status and who have never been linked to care, or have declined ART, or were lost to follow-up (World Health Organization, 2021). In this regard, HB-HCT constitutes an opportunity to re-refer those people to HIV care. This is particularly relevant in the study area, where only 53% of diagnosed PLHIV were actively in care at baseline (i.e., the ‘second 95’, Larmarange et al. (2019)), and where the rate of linkage to care remained sub-optimal throughout the TasP trial (Plazy et al., 2016). In TasP, we estimated the cost per appropriate referral to HIV care to be only $269.2 (SD 279.0).

Among the four UTT trials, the cost-effectiveness of repeated HB-HCT has only been estimated in the PopART trial in Zambia and South Africa (Thomas et al., 2021). This study found that combination HIV prevention, including universal and repeated HB-HCT, would be cost-effective at thresholds ≥$800 per disability-adjusted life year (DALY) averted compared with facility-based care provision (the standard-of-care). However, simulated annual costs were shown to accumulate to a considerable amount when projecting coverage for the entire population.

### 4.2 Economies of scale in repeated HB-HCT

Using a dynamic system GMM approach that allowed us to obtain unbiased short- and long-run estimates of economies of scale, this paper revealed the presence of cost savings from scaling up HB-HCT activities. These significant cost savings could not have been captured by the constant average cost projections that are generally reported in existing costing studies of HB-HCT.

Specifically, we found that a 1% increase in the scale of HB-HCT activities – measured as the number of contacts made by an HIV counsellor for HB-HCT – led to a 0.27% decrease in the average cost of HB-HCT activities. Overall, our results show that the marginal cost of HB-HCT (i.e., the cost of an additional contact for HB-HCT) tends to decrease when the population coverage increases, highlighting opportunities for cost savings from scaling up HB-HCT activities. Moreover, we provide evidence for a significant long-run relationship between scale and average cost. Once the HB-HCT programme becomes fully operational, a 1% increase in the scale would lead to a 0.36% decrease in the average cost. Here, we assume that, from a long-run perspective, the public provider would operate at the minimum of the short-run average cost curve (Lépine et al., 2015), that is, at the lowest possible average cost per contact for HB-HCT.

Our results could help to inform recommendations regarding the optimal operational size for an HB-HCT cluster. As the direction of causality matters for policy, any such recommendation may be drawn only if causality runs mainly from volume to outcome (Gaynor et al., 2005). Our analysis provides evidence for a causal effect of scale on average cost. Accordingly, it can be drawn from our results that increasing the size of HB-HCT clusters to expand coverage would result in greater cost savings. In other words, cost savings from economies of scale would be greater in the presence of fewer HB-HCT geographical clusters but larger in terms of population coverage. In TasP, the average size of an HB-HCT cluster was 1307 adult residents. Hence, the average costs would likely be reduced by increasing the population coverage beyond this average size (all other things being equal).

We might expect these economies of scale to be driven by (i) the presence of fixed costs, which are incurred only at the beginning of each cluster, and (ii) learning-by-doing (i.e., when today's scale affects both today's and future average costs), since the cost breakdown provided in Table 2 indicates that personnel costs represented the highest cost category (57.9% of total costs). We ruled out the hypothesis that economies of scale were driven by lower input prices, since the Africa Health Research Institute had no bargaining power in input price negotiations.

To our knowledge, the two studies by Lépine et al., 2015, Lépine et al., 2016 in India, are the only previous studies estimating (unbiased) economies of scale in HIV-related activities. Our estimates of economies of scale (between −0.239 and −0.268) were lower than those in Lépine et al. (2015) when considering programme costs (between −0.670 and −0.823). Any direct comparison should be made with caution, as the activities and contexts of the two studies were quite different. Lépine et al., 2015, Lépine et al., 2016 focused on primary prevention activities (including prevention information provision, condom and lubricant distribution, and referral for the management of sexually transmitted infections), while we investigated secondary prevention using HB-HCT.

Finally, we showed that these economies of scale are reduced – although still present – when the quantity-quality trade-off in HB-HCT activities tilts in favour of quality. Besides leading to higher average costs per contact by an HIV counsellor, the higher the contact quality – measured as the proportion of HIV tests performed among all contacts eligible for an HIV test – the lower the cost savings when scaling up HB-HCT.

### 4.3 Study limitations

Our study has several limitations. First, estimations were based on a single clinical trial conducted in rural South Africa, which may limit the generalizability of the results. In particular, we cannot fully exclude the presence of diseconomies of scale in other settings and/or at the national scale. Nevertheless, the TasP trial was conducted in a real-world large-scale healthcare delivery setting, and we disentangled time and resources devoted to research from those devoted to HB-HCT activities, considering only the latter in all our estimations.

Second, we may have overestimated the costs of HB-HCT, which is a common drawback of clinical trial-based cost estimations. For instance, one might expect the personnel costs to be lower in real-world settings. However, we do not expect this potential overestimation to differ across clusters and months, and therefore it should not have affected economies of scale estimates. It is important to highlight that we did not account for the cost of staff training. This omission may have led to an underestimation of both mean costs and, as staff training is likely to occur at the beginning of HB-HCT activities, scale economies.

Finally, our scale variable for the estimation of economies of scale was the number of contacts by an HIV counsellor for HB-HCT, while other expected outcomes were the number of HIV tests performed, the number of positive HIV tests that eventually led to a referral to a clinic for immediate ART initiation, and, finally, HIV incidence in the population. However, we considered the number of contacts as the scale variable, since HB-HCT programme implementers may only have a direct influence on the number (and the quality) of contacts, which is a precondition to meet the other outcomes. It is also important to underline that our analysis accounted for the quality of HB-HCT activities, defined as the proportion of HIV tests performed among all contacts eligible for an HIV test.

## 5 Conclusions

The ANRS 12249 TasP trial in South Africa allowed us to estimate the cost of large-scale, universal, and repeated HB-HCT. Our results provide critical information for planners and decision-makers when evaluating the economic sustainability of such a strategy and developing guidelines, especially in contexts characterised by limited human and material resources and the emergence of new diseases. We also revealed the presence of economies of scale when this intervention is scaled up. Nevertheless, although scaling up activities was shown to reduce the mean cost of HB-HCT, there is a risk that this gain is made at the expense of the quality of HB-HCT activities. More specifically, in our study, the negative (causal) effect of scaling up HB-HCT activities on mean costs, while still present, was mediated by the contact quality.

Overall, the identification of these economic efficiency gains may help inform decisions on the implementation of universal and repeated HB-HCT. The presence of important cost savings from economies of scale in HB-HCT – which had been viewed as a costly strategy – should be considered when compared with other strategies (including facility-based (i.e., the standard-of-care), community-based mobile, and hybrid mobile HIV counselling and testing).

## ANRS 12249 TasP Study Group

*South Africa* Till Barnighausen, Kobus Herbst, Collins Iwuji, Thembisa Makowa, Kevi Naidu, Nonhlanhla Okesola, Tulio de Oliveira, Deenan Pillay, Tamsen Rochat, Frank Tanser, Johannes Viljoen, Thembelihle Zuma (Africa Health Research Institute [previously Africa Centre for Population Health, University of KwaZulu-Natal], KwaZulu-Natal, Durban). Frank Tanser, Nuala McGrath (School of Nursing and Public Health, University of KwaZulu-Natal, KwaZulu-Natal, Durban). Tulio de Oliveira (Nelson R Mandela School of Medicine, College of Health Sciences, University of KwaZulu-Natal, KwaZulu-Natal, Durban). *France* Eric Balestre, Francois Dabis, Sophie Karcher, Joanna Orne-Gliemann, Melanie Plazy, Melanie Prague, Rodolphe Thiebaut, Thierry Tiendrebeogo (ISPED, Centre INSERM U1219 Bordeaux Population Health, Universite de Bordeaux, Bordeaux). Sylvie Boyer, Marwân-al-Qays Bousmah, Hermann Pythagore Pierre Donfouet, Andrea Gosset, Laura March, Camelia Protopopescu, Bruno Spire (INSERM, UMR1252 SESSTIM, Universite Aix Marseille, Marseille). Joseph Larmarange, Maxime Inghels, Hassimiou Diallo (Centre Population et Developpement UMR 196, Universite Paris Descartes, Institut de Recherche pour le Developpement, Paris). Vincent Calvez, Anne Derache, Anne-Genevieve Marcelin (AP-HP, Virology, Hopital Pitie-Salpetriere, INSERM-Sorbonne Universites, UPMC Univ Paris 06, UMR-S 1136, Paris). Rosemary Dray-Spira, France Lert, Kamal El Farouki (INSERM U1018, CESP, Epidemiology of Occupational and Social Determinants of Health, Villejuif). Marie-Laure Chaix (EA 3620, Universite Paris-Descartes, Laboratoire de Virologie, Hopital Necker-Enfants Malades, AP-HP, Paris). Brigitte Bazin, Claire Rekacewicz (sponsor representatives; ANRS, Paris). *UK* Collins Iwuji, John Imrie (Department of Infection and Population Health, University College London, London). Deenan Pillay (Division of Infection and Immunity, University College London, London). Nuala McGrath (Department of Epidemiology and Public Health, University College London, London). Richard Lessells (Department of Clinical Research, London School of Hygiene & Tropical Medicine, London). Collins Iwuji (Department of Global Health and Infection, Brighton and Sussex Medical School, University of Sussex, Brighton). Nuala McGrath (Academic Unit of Primary Care and Population Sciences, and Department of Social Statistics and Demography, University of Southampton, Southampton). Colin Newell (Academic Unit of Human Development and Health, University of Southampton, Southampton). Marie-Louise Newell, (Academic Unit of Human Development and Health, and Global Health Research Institute, University of Southampton, Southampton). *Switzerland* Alexandra Calmy (Service des Maladies Infectieuses, HIV Unit, Hopitaux Universitaires de Geneve, Geneva). *USA* Kenneth Freedberg (Massachusetts General Hospital, Harvard Medical School, Harvard University, Boston, MA). Till Barnighausen (Department of Global Health and Population, Harvard School of Public Health, Harvard University, Boston, MA). *Netherlands* Jan Hontelez (Department of Public Health, Erasmus MC, Erasmus University Medical Center Rotterdam, Rotterdam). *Germany* Till Barnighausen, Jan Hontelez (Institute of Public Health, Faculty of Medicine, Heidelberg University, Heidelberg).

## CRediT author statement

Marwân-al-Qays Bousmah: Conceptualization, Methodology, Formal analysis, Visualization, Writing – original draft, Writing – review & editing. Collins Iwuji: Conceptualization, Project administration, Investigation, Writing – review & editing. Nonhlanhla Okesola: Project administration, Writing – review & editing. Joanna Orne-Gliemann: Conceptualization, Project administration, Writing – review & editing. Deenan Pillay: Funding acquisition, Conceptualization, Writing – review & editing. François Dabis: Funding acquisition, Conceptualization, Writing – review & editing. Joseph Larmarange: Conceptualization, Software, Data curation, Writing – review & editing. Sylvie Boyer: Conceptualization, Supervision, Methodology, Writing – review & editing.

## Funding

The ANRS 12249 TasP trial was sponsored by the French National Agency for AIDS and Viral Hepatitis Research (ANRS; grant number, 2011-375), and funded by the ANRS, the Deutsche Gesellschaft für Internationale Zusammenarbeit (GIZ; grant number, 81151938), and the Bill & Melinda Gates Foundation through the 3ie Initiative. The Africa Health Research Institute (previously Africa Centre for Population Health, University of KwaZulu Natal, South Africa) receives core funding from the Wellcome Trust, which provides the platform for the population-based and clinic-based research at the centre.

## Declaration of competing interest

Collins Iwuji has received honoraria for consulting services and research grants unrelated to this work from Gilead Sciences. All other authors report no conflict of interest.
