# Supplementary material for: Costs and economies of scale in repeated home-based HIV counselling and testing: Evidence from the ANRS 12249 Treatment as Prevention trial in South Africa
Source: Soc Sci Med. 2022 Jul;305:115068. doi: 10.1016/j.socscimed.2022.115068 (PMC9214548; doi:10.1016/j.socscimed.2022.115068)
Supplement: Multimedia component 1 [file mmc1.docx]

**Electronic Supplementary Material**

This online resource has been provided by the authors to give readers additional information about their work.

Supplement to: *Costs and economies of scale in repeated home-based HIV counselling and testing: Evidence from the ANRS 12249 Treatment as Prevention trial in South Africa*

**Contents:**

**Page 2:** Figure A1. Total number of residents aged ≥16 years registered, of contacts made for HB-HCT, of contacts eligible for an HIV test, of HIV tests performed, of positive HIV tests, of new HIV diagnoses, and of appropriate referrals to HIV care, per month, over the trial period

**Page 3-4:** Table A1. Home-based HIV counselling and testing and costs (in US$ 2016) per year and over the whole study period (March 2012-April 2016)

**Page 5:** Table A2. Cost breakdown per year and over the whole study period (March 2012-April 2016)

**Figure A1. Total number of residents aged ≥16 years registered, of contacts made for HB-HCT, of contacts eligible for an HIV test, of HIV tests performed, of positive HIV tests, of new HIV diagnoses, and of appropriate referrals to HIV care, per month, over the trial period**


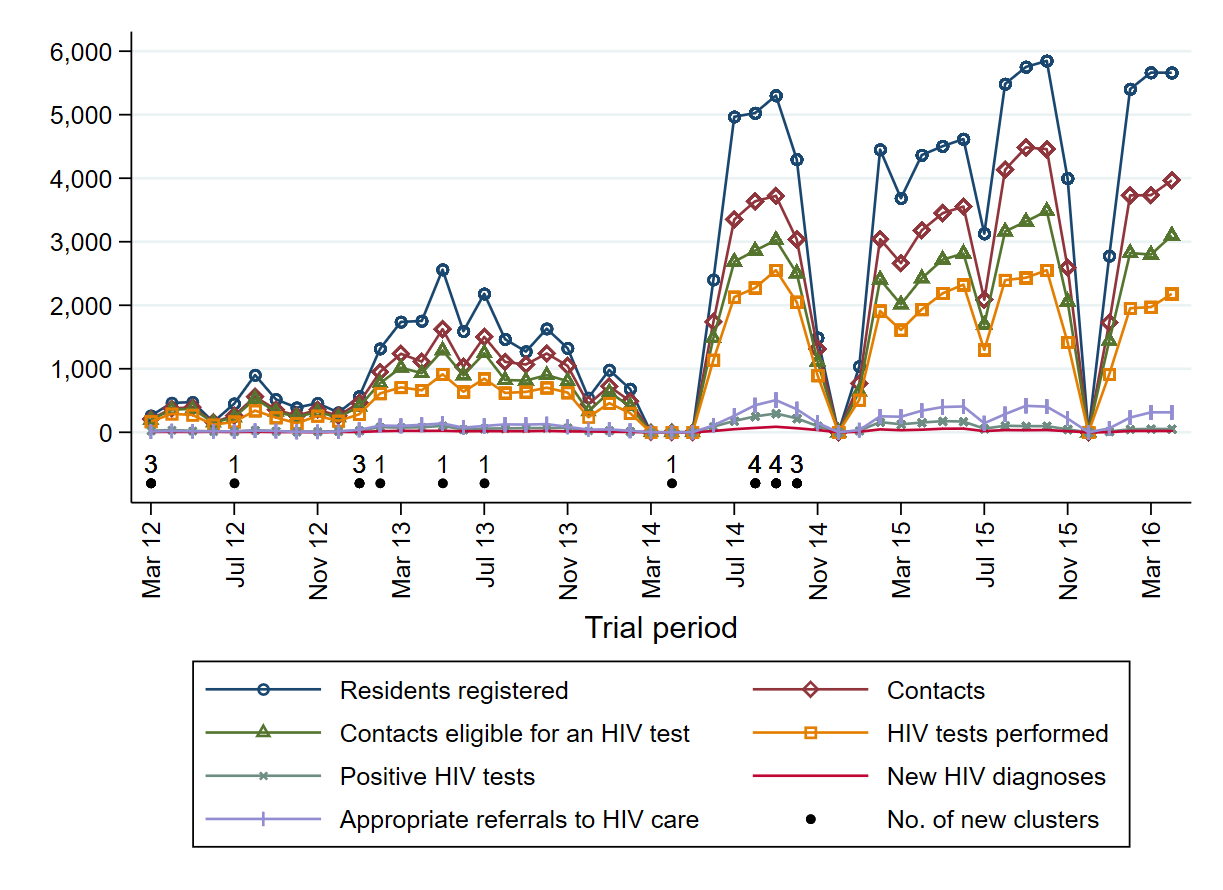


**Table A1. Home-based HIV counselling and testing and costs (in US$ 2016) per year and over the whole study period (March 2012-April 2016)**

|  | **2012**  **(March-December)** | **2013** | **2014** | **2015** | **2016**  **(January-April)** | **Whole period** |
| --- | --- | --- | --- | --- | --- | --- |
| **Registered clusters** | 4 | 10 | 22 | 22 | 22 | 22 |
| **(a) No. of individuals registered** | 3,399 | 11,651 | 22,796 | 25,886 | 20,967 | 28,347 |
| **(b) No. of contacts for HB-HCT** | 3,194 | 12,829 | 18,013 | 34,418 | 13,157 | 81,611 |
| **(c) No. of contacts eligible for an HIV test** | 2,960 | 10,231 | 14,715 | 26,737 | 10,161 | 64,804 |
| **(d) No. of HIV tests performed** | 2,169 | 7,482 | 11,801 | 20,575 | 7,019 | 49,046 |
| **(e) No. of positive HIV tests** | 203 | 754 | 1,175 | 1,212 | 166 | 3,510 |
| **(f) No. of new HIV diagnoses** | 51 | 208 | 339 | 383 | 65 | 1,046 |
| **(g) No. of appropriate referrals to HIV care** | 147 | 1,180 | 1,884 | 3,141 | 922 | 7,274 |
| **Total cost** | 217,881.7 | 301,216.0 | 497,860.4 | 680,100.6 | 281,265.0 | 1,978,323.6 |
| **Cost per contact** |  |  |  |  |  |  |
| **Mean** | 68.2 | 23.5 | 27.6 | 19.8 | 21.4 | 24.2 |
| **(SD)** | (24.9) | (8.4) | (10.8) | (8.7) | (6.3) | (13.7) |
| **Median** | 57.8 | 21.1 | 23.5 | 18.0 | 19.3 | 19.8 |
| **(IQR)** | (56.8–77.9) | (19.4–25.9) | (19.9–27.7) | (15.1–20.2) | (18.5–19.9) | (17.9–23.7) |
| **Cost per contact eligible for an HIV test** |  |  |  |  |  |  |
| **Mean** | 73.6 | 29.4 | 33.8 | 25.4 | 27.7 | 30.5 |
| **(SD)** | (26.7) | (11.0) | (12.3) | (10.5) | (7.3) | (15.4) |
| **Median** | 64.1 | 27.3 | 30.7 | 22.7 | 25.7 | 25.4 |
| **(IQR)** | (59.3–87.5) | (22.9–32.5) | (24.8–37.7) | (19.5–28.2) | (23.2–27.1) | (22.4–31.3) |
| **Cost per HIV test performed** |  |  |  |  |  |  |
| **Mean** | 100.4 | 40.2 | 42.1 | 33.0 | 40.1 | 40.3 |
| **(SD)** | (35.0) | (14.7) | (16.2) | (13.6) | (12.5) | (20.7) |
| **Median** | 82.4 | 35.7 | 36.8 | 28.8 | 35.8 | 34.6 |
| **(IQR)** | (76.0–127.9) | (33.8–41.7) | (31.0–42.3) | (26.2–34.8) | (34.4–38.7) | (28.8–40.5) |
| **Cost per positive HIV test** |  |  |  |  |  |  |
| **Mean** | 1,051.0 | 394.5 | 418.8 | 546.6 | 1,532.2 | 546.9 |
| **(SD)** | (735.9) | (337.6) | (249.9) | (451.6) | (1,913.4) | (620.2) |
| **Median** | 776.1 | 314.3 | 318.2 | 404.6 | 1,139.3 | 376.0 |
| **(IQR)** | (666.0–1,113.5) | (228.2–393.2) | (254.1–496.3) | (323.0–570.9) | (787.9–1,691.5) | (275.5–621.0) |
| **Cost per new HIV diagnosis** |  |  |  |  |  |  |
| **Mean** | 3,750.9 | 1,323.6 | 1,432.5 | 1,636.2 | 2,975.3 | 1,694.3 |
| **(SD)** | (1,966.7) | (793.3) | (941.6) | (1,600.7) | (2,765.7) | (1,527.8) |
| **Median** | 3,230.7 | 1,078.5 | 1,183.8 | 1,173.0 | 2,383.8 | 1,207.6 |
| **(IQR)** | (2,493.6–4,397.5) | (956.1–1,710.3) | (859.0–1,596.8) | (888.2–1,728.9) | (1,374.0–3,575.6) | (914.9–1,906.7) |
| **Cost per appropriate referral to HIV care** |  |  |  |  |  |  |
| **Mean** | 1,460.3 | 253.2 | 260.9 | 214.1 | 304.4 | 269.2 |
| **(SD)** | (769.3) | (190.8) | (182.7) | (182.8) | (242.7) | (279.0) |
| **Median** | 1,189.0 | 173.9 | 184.1 | 172.0 | 241.6 | 185.2 |
| **(IQR)** | (993.7–2,036.5) | (168.2–281.0) | (140.3–289.6) | (134.6–229.4) | (206.3–288.1) | (150.8–273.6) |
| Notes: Monetary amounts are provided in US$ (year 2016 values).  Definitions: (a) number of residents aged ≥16 years registered in the ANRS 12249 TasP trial (i.e., the target population eligible for HB-HCT, whose enumeration was updated at each survey round to account for in- and out-migration, individuals turning 16, and deaths), (b) number of contacts made by HIV counsellors to offer HB-HCT, (c) number of contacts eligible for an HIV test according to trial procedures (i.e., all contacts except those who self-reported being HIV-positive to the field worker), (d) number of rapid HIV tests performed, (e) number of positive rapid HIV tests, (f) number of new HIV diagnoses (contacts newly diagnosed as HIV positive, taking into account previous contacts and records in local governmental clinics), and (g) number of appropriate referrals to HIV care (i.e., contacts where the person was ascertained HIV positive through rapid testing or self-report, and was not currently in HIV care in a local governmental clinic or a trial clinic).  Abbreviations: HB-HCT=home-based HIV counselling and testing. SD=standard deviation. IQR=interquartile range. | | | | | | |

**Table A2. Cost breakdown per year and over the whole study period (March 2012-April 2016)**

|  | **2012**  **(March-December)** | **2013** | **2014** | **2015** | **2016**  **(January-April)** | **Whole period** |
| --- | --- | --- | --- | --- | --- | --- |
| **Recurrent costs** | 171,851.6 | 223,974.6 | 429,093.8 | 559,856.0 | 237,257.3 | 1,622,033.3 |
| **(% of total costs)** | (78.9) | (74.4) | (86.2) | (82.3) | (84.4) | (82.0) |
| - Personnel | 126,307.6 | 139,153.0 | 279,560.5 | 433,568.4 | 166,594.8 | 1,145,184.3 |
| (% of total costs) | (58.0) | (46.2) | (56.2) | (63.8) | (59.2) | (57.9) |
| - Transport | 34,193.5 | 47,411.8 | 89,931.3 | 24,677.1 | 37,172.6 | 233,386.3 |
| (% of total costs) | (15.7) | (15.7) | (18.1) | (3.6) | (13.2) | (11.8) |
| - Communication | 2,171.5 | 6,591.5 | 12,592.9 | 21,737.2 | 6,794.0 | 49,887.2 |
| (% of total costs) | (1.0) | (2.2) | (2.5) | (3.2) | (2.4) | (2.5) |
| - HIV tests and supplies | 9,179.1 | 30,818.2 | 47,009.1 | 79,873.3 | 26,695.8 | 193,575.5 |
| (% of total costs) | (4.2) | (10.2) | (9.4) | (11.7) | (9.5) | (9.8) |
| **Capital costs** | 46,030.1 | 77,241.4 | 68,766.5 | 120,244.5 | 44,007.7 | 356,290.4 |
| **(% of total costs)** | (21.1) | (25.6) | (13.8) | (17.7) | (15.6) | (18.0) |
| **Total costs** | 217,881.7 | 301,216.0 | 497,860.4 | 680,100.6 | 281,265.0 | 1,978,323.6 |
| Notes: Monetary amounts are provided in US$ (year 2016 values). | | | | | | |
